# Student response to reports of unprofessional behavior: assessing risk of subsequent professional problems in medical school

**DOI:** 10.1080/10872981.2018.1485432

**Published:** 2018-06-18

**Authors:** Michael A. Ainsworth, Karen M. Szauter

**Affiliations:** a Department of Internal Medicine and Senior Associate Dean for Educational Performance, University of Texas Medical Branch, Galveston, TX, USA; b Department of Internal Medicine and Assistant Dean, Educational Affairs, University of Texas Medical Branch, Galveston, TX, USA

**Keywords:** Professionalism, medical students, academic risk, early concern note, learning environment

## Abstract

**Background:** An early concern note (ECN) program is used by some medical schools to identify, counsel, and intervene when students exhibit unprofessional behavior. Student maturity, insight, propensity for reflection, and receptiveness to feedback have been suggested as predictors of future behavior. **Objective**: We hypothesized that (a) classifying students with a first ECN based on their response to the report would identify students at risk of repeat ECNs better than the action that prompted it and (b) receipt of multiple ECNs would identify students at risk of adverse academic events.

**Design:** For this study, 459 ECNs were classified based on students’ (1) recognition that their behavior was inappropriate and (2) acceptance of responsibility for the behavior. Student academic progress and receipt of subsequent ECNs were tracked.

**Results:** Students who recognized their behavior was inappropriate and accepted responsibility after an initial ECN received subsequent ECNs at lower rates (14–19%) than students who disagreed with the significance of their behavior or were resistant to accepting responsibility (36–59%). Students with limited insight and adaptability appeared to be at highest risk. Seventy-one percent of students with three or more ECNs encountered adverse academic events during enrollment.

**Conclusion:** Student reactions to reports of unprofessional behavior may be useful as a tool to help assess risk of recurrent lapses. Students with diminished capacity to recognize behaviors as unprofessional or accept responsibility for them appear to be at highest risk for additional adverse academic and professionalism events while in medical school.

## Introduction

Appreciation for the importance of professionalism in the practice of medicine is ubiquitous [1,2]. Medical school faculty face the challenge of defining the behaviors through which student professionalism is judged and the standards of performance to which learners are held. Ideally, students should learn about professional behavior expectations, see those behaviors reinforced by role models in a supportive environment, have their behaviors measured appropriately, and receive guidance when they struggle [3]. Because learning environments are often not ideal, professionalism lapses can go unnoticed, undocumented, and unaddressed. Previous work supports a relationship between medical school performance and future unprofessional behavior [4,5]. Less is known about prospectively identifying learners at highest risk of repetitive problems while in medical school.

Characterizing a learner’s professional attributes does not lend itself to a single point-in-time measurement. Better longitudinal assessments are needed [6]. It is especially useful to identify students most at risk of serious or repeated deficiencies at the earliest possible time and to distinguish them from students who experience isolated transgressions. While objective structured clinical exams, multiple mini-interviews, and other techniques hold promise for identifying some at-risk behaviors, longitudinal tools and techniques are needed that can be used across the training continuum [7,8]. A variety of processes are used throughout North American medical schools to identify or track professionalism issues, including incident-based reports, courses on professionalism, and peer assessment [9]. Interpreting the growing literature on professionalism is complicated by various classification systems used to group types of behaviors deemed as unprofessional [10,11].

Our medical school is a large (230 students/year) institution functioning as part of a public, multidisciplinary health science campus. The curriculum follows a conventional ‘2 + 2’-year sequence but differs from many schools in its heavy emphasis on small-group problem-based learning. The early concern note (ECN) in our school, modeled after similar initiatives at other institutions [12], has been used for 17 years as part of a comprehensive approach to creating a professional environment. Initially intended for course and clerkship directors, its use has been expanded so that any faculty or staff member who has contact with a medical student may report on his or her behavior. The ECN instrument focuses on representative behaviors (Table 1) developed through a school-wide consensus-building process. Three major categories of physician performance are represented—integrity and reliability; motivation and insight; and compassion and respect.

**Table 1.** Early concern note behaviors (modeled after Papadakis [12]).

Integrity and personal responsibility
● Fails to fulfill responsibilities reliably
● Misrepresents or falsifies actions and/or information
● Fails to accept responsibility for actions
● Fails to respect patient confidentiality
● Uses his/her professional position to take advantage of a patient
Motivation to pursuit of excellence and insight for self-improvement
● Inadequate personal commitment to honoring the needs of patients
● Resistant or defensive in accepting criticism
● Unaware of his/her limits
● Resists changes based on feedback
● Seeks minimally acceptable level of effort
Personal interactions of compassion and respect
● Inadequate rapport or empathy
● Fails to function and interact appropriately
● Insensitive to the needs, feelings, and wishes of others
● Uses demeaning or disrespectful language about others
● Abusive of arrogant under stress
● Fails to maintain professional appearance and attire

The principles guiding the use of ECNs may be more important than the words on a reporting form [13]. At our institution, the ECN is designed to be a supportive ‘early-warning’ device that serves several educational functions: a trigger for counseling students who exhibit problematic behavior, a longitudinal tracking system of such behaviors, and the basis for a graded (progressive) program of counseling and intervention. Faculty, staff, and students are educated about the purpose of the ECN each year. Faculty and staff are encouraged to use it to report behaviors of concern, including events they may deem insufficient to impact official evaluations or grades. The program operates as follows:

- Upon receipt of a student’s first ECN, a copy is sent to the student with a reminder of the purpose of the ECN process and instructions to review the report prior to meeting with the project’s director. A first ECN results in a confidential discussion with the student and informal counseling is provided, but no punitive measures result. The first ECN is not part of the student’s academic record and is not shared with any other parties, including faculty who may encounter the student in a future course or clerkship.
- A second ECN results in a confidential discussion between the student and a larger group of education deans. Additional counseling is provided, with attention to commonalities that may appear between the two ECNs. As is the case for a first ECN, no punitive measures result, and the ECN is not part of the academic record. Exceptions to confidentiality of first and second ECNs occur when the student is felt to be a danger to him-/her-self or others.
- A third (or subsequent) ECN results in the student appearing before the school’s academic progress committee and the student is subject to official action, ranging from mandatory counseling to academic consequences, up to and including dismissal. These actions (and ECNs) become part of both the academic record and the student’s Medical Student Performance Evaluation letter sent to postgraduate training programs.

Our approach to addressing recipients of three or more ECNs developed over time and reflects our concern with this limited group of students. Early observations demonstrated that students with one or two ECNs during enrollment have academic outcomes indistinguishable from students without an ECN. Prospectively identifying the subgroup of students who will progress to three or more ECNs remained a challenge. Previous authors have suggested that student maturity, insight, propensity for reflection, or receptiveness to feedback might be related to future behavior [14–16]. The receipt of an ECN may be characterized as a ‘stressful’ event which asks a student to reflect on behavioral change and accept or reject counseling and feedback.

Our previous work failed to find an association between the specific ECN behavior reported (e.g., attendance problem and poor group interaction) and progression to additional ECNs. We hypothesized that student response to being confronted with an ECN would better identify students at-risk. To study this, we classified students based on their response to the ECN and tracked students’ receipt of subsequent reports. Additionally, we tracked academic performance during the study period.

## Materials and methods

ECNs are submitted electronically. The person submitting the report notes that the category(ies) of behavior and provides a detailed description of behavior(s) triggering the report. Beginning in 2010, student response to receipt of an ECN was documented. Responses were classified based on (1) whether (or not) the student recognized and acknowledged his or her behavior as inappropriate and (2) whether (or not) the student accepted responsibility for the behavior.

This report includes data on students receiving a first ECN during a seven-year period (October 2010 through December 2017), any subsequent ECNs, and academic performance history.

The study used a correlational design. Classification of student responses was performed by one of the authors (MA) immediately after meeting with each student. Table 2 summarizes the four classifications and sample student comments. Each meeting began by providing the student another copy of the ECN and asking the student ‘what would you like to tell me about this report?’ Students were placed in classification (class) 1 only if they spontaneously (without further prompting) volunteered that they recognized their behavior was inappropriate and accepted responsibility for their actions. Students were placed in class 2 if, after prompting during the discussion, they ultimately acknowledged that the behavior was inappropriate and accepted responsibility. Class 3 represented students who, after discussion, were able to recognize their errors, but deflected personal responsibility to others, agreeing reluctantly to comply. Class 4 included students who demonstrated neither recognition nor acceptance of responsibility. Reliability of classifying student reactions was assessed by a secondary review of a subset (*n* = 24) of ECNs across all four response classes by two additional independent raters. These raters, blinded to student identity and the original classification, received the ECN and a written summary of the corresponding student responses. Level of agreement across raters was compared and demonstrated 92% interrater agreement. Academic performance was assessed by review of student transcripts; frequency of adverse academic events (defined as required course and clerkship grades of less than ‘Pass’) was recorded for each student.

**Table 2.** Classification of student response to an early concern note.

	Class 1	Class 2	Class 3	Class 4
Recognition	Spontaneously recognized that behavior was inappropriate	Recognized error when brought to their attention	Receptive to recognizing inappropriate nature of their behavior after discussion	Remained resistant to recognizing inappropriate action or denied it occurred
Responsibility	Spontaneously accepted responsibility	Accepted responsibility when described to them	Remained resistant to accepting responsibility but agreed to comply anyway	Rejected responsibility
Sample comment	‘I forgot to make a note of that schedule change; I’m sorry I missed it and embarrassed I messed up.’	‘I had no idea they took attendance at orientation so seriously. It won’t happen again.’	‘I realize the course wants it done a certain way. I still think it’s unnecessary, but I’ll do what they say.’	‘The course is blowing it way out of proportion. I spoke my mind and they are taking it out of context.’

We used Fisher’s exact test to determine whether there were differences in the proportion of students with repeat ECNs based on the classification of student reactions. Significance was defined as a difference measured at the *p* < 0.05 level.

Data for this study were derived from information collected as a part of the school’s standard educational practices. This study was approved by the University of Texas Medical Branch Institutional Review Board. Permission for use of curriculum data was obtained from the School of Medicine Curriculum Committee [17].

## Results

Since inception of the ECN program at our institution in 2000, over 700 ECNs have been submitted. The study period includes data from 459 ECNs submitted on 344 students, specifying 644 behaviors. The ECNs, which could report behaviors in more than one category, included issues of integrity and personal responsibility (418 behaviors; 65% of the total), motivation to the pursuit of excellence and insight for self-improvement (125; 19%), and personal interactions of compassion and respect (101; 16%). Nearly equal numbers of ECNs were received by Year 1 and 2 students (237; 52%) and Year 3 and 4 students (222; 48%), and the distribution of reported behaviors across the three categories was similar for pre-clerkship (Year 1 and 2) and clinical (Year 3 and 4) students. Most submissions (289; 63%) were from course and clerkship directors or other faculty, with the remainder coming from staff members (most often the course and clerkship coordinator staff). The majority of ECNs (263; 57%) reported behaviors directly observed; the others (196; 43%) represented behaviors reported to the submitter by a third party (typically to course or clerkship personnel from a person working directly with a student).

Classification of student responses to ECNs and frequency of progression to subsequent ECNs are summarized in Tables 3 and 4. Table 3 sorts student responses after a first ECN and frequency of progression to subsequent ECNs; Table 4 represents the responses of students after a second ECN and the frequency of receipt of additional (third or greater) ECNs.

**Table 3.** Classification of *student responses following receipt of a first ECN* and rate of progression to subsequent ECNs.

ECN recipients grouped by response to first ECN *n* = 344				
	*Knew they erred* Class 1 *n* (%)	*Naive* Class 2 *n* (%)	*Resistant but able to Adapt* Class 3 *n* (%)	*Unable to adapt* Class 4 *n* (%)
ECN #1	162 (47.1)	78 (22.7)	70 (20.3)	34 (9.9)
				
Progressed to second ECN	23 of 162 (14.2)	15 of 78 (19.2)^a^	25 of 70 (35.7)^b^	20 of 34 (58.8)^c^
				
Progressed to third ECN	2 of 162 (1.2)	1 of 78 (1.3)†	13 of 70 (18.6)††	5 of 34 (14.7)†††

^a^FET = 0.2646 (nonsignificant) compared to class 1.

^b^FET = 0.0273 (*p* < 0.05) compared to class 2.

^c^FET = 0.0349 (*p* < 0.05) compared to class 3 and <0.0001 (*p* < 0.05) compared to class 2.

FET < 0.0001 (*p* < 0.05) for combined class 1 + 2 compared to combined class 3 + 4.

†FET = 1 (nonsignificant) compared to class 1.

††FET = 0.0003 (*p* < 0.05) compared to class 2.

†††FET = 0.7845 (nonsignificant) compared to class 3 and 0.0096 (*p* < 0.05) compared to class 2.

FET < 0.0001 (*p* < 0.05) for combined class 1 + 2 compared to combined class 3 + 4.

**Table 4.** Classification of *student responses following receipt of a second ECN* and rate of progression to subsequent ECNs.

ECN recipients grouped by response to second ECN *n* = 83				
	*Knew they erred* Class 1 *n* (%)	*Naive* Class 2 *n* (%)	*Resistant but able to adapt* Class 3 *n* (%)	*Unable to adapt* Class 4 *n* (%)
ECN #2	11 (13.3)	15 (18.1)	25 (30.1)	32 (38.6)
				
Progressed to third ECN	0 of 11 (0.0)	2 of 15 (13.3)*	6 of 25 (24.0)**	13 of 32 (40.6)***

*FET = 0.4923 (nonsignificant) compared to class 1.

**FET = 0.6857 (nonsignificant) compared to class 2.

***FET = 0.2597 (nonsignificant) compared to class 3 and 0.0944 (nonsignificant) compared to class 2.

FET = 0.0142 (*p* < 0.05) for combined class 1 + 2 compared to combined class 3 + 4.

A total of 240 (70%) first-ECN students had responses placed in class 1 or 2. These students might be conceptually viewed as individuals who spontaneously knew that they erred (class 1) or were initially naive about professional expectations (class 2). The common feature across the two groups is that they appeared to have no intent to violate professional norms. These two groups progressed to a second ECN at low rates (14% and 19%, respectively).

A total of 104 (30%) first-ECN students had responses placed in class 3 or 4, which appears to represent students more challenged by professional expectations. As shown in Table 3, the subset of students in class 3 (initially disagreed but expressed willingness to adapt) represented 20% of the study population, and these students progressed to a second ECN at a statistically significant higher rate than class 2 (36% vs. 19%). The 10% of students in class 4 (remained resistant to recognizing the behavior or accepting responsibility) progressed at an even higher rate (59%). The findings were similar for progression of these students to a third ECN. The response classifications for students after receiving a second ECN (Table 4) reached statistical significance for classes 1 and 2 combined compared to classes 3 and 4 combined. Neither year of medical school nor category of behavior (integrity, motivation, and interactions) differed across students in the four classes. Behaviors reported by third parties were not different from those directly reported, most likely because the third parties (course/clerkship personnel) were merely conduits for ECN submission by the faculty who directly observed the behavior.

Adverse academic events are relatively uncommon in the general student population, representing 1.3% of preclinical course grades and 2.2% of clerkship grades in academic year 2016–2017. In contrast, such events were recorded by 15 of 21 (71%) of students with three or more ECNs, primarily based on performance on traditional academic measures of knowledge such as multiple-choice-question examinations. Of those 15 students, 6 have been dismissed for a range of academic and professional deficiencies. Most (40/52; 77%) of the ECNs received by those 15 students occurred outside the courses during which an academic deficiency was reported. No pattern was discernable whether ECNs occurred prior to or after a student’s academic difficulty.

## Discussion

At our institution, most students complete their studies without a report of unprofessional behavior. The vast majority of ECNs that are submitted represent students who receive only one or two ECNs during their medical school career—students whose academic outcomes are indistinguishable from students without an ECN. Yet, some students progress to additional reports, and this subgroup was the focus of this report.

Our findings support our hypothesis that student reaction to a report of unprofessional behavior identifies individuals with a high risk of subsequent professional lapses. In our student population, those classified as having insight into their behavior and no recognizable intent to behave in an unprofessional manner (classes 1 and 2) progressed to a subsequent ECN at a statistically lower rate than those who might be seen as more challenged by professional expectations (classes 3 and 4). Such reactions can be a reflection of the student’s maturity, insight, and adaptability to the professional challenges faced by all physicians, making these relevant skills to cultivate [16]. Students with diminished capacity to adapt to behavioral expectations or to accept responsibility for them appear to be at highest risk of future reports of unprofessional behavior, reinforcing the importance of insight in professional performance [18–20].

Not all ECNs necessarily reflect a student’s underlying character. The observed behaviors triggering an ECN at our institution often reflected a range of underlying problems. For example, ‘a pattern of frequently arriving late’ has included underlying factors as diverse as failure to set a wake-up alarm, sleep disturbance from a newborn child at home, ignorance (or dismissal) of professional norms, declining interest in medicine as a career, depression, burnout, or substance abuse [21,22]. In such cases, the behavior might represent a transient, self-limited, or treatable condition or provide an opportunity to educate the student. Thus, the value of an ECN does not always lie in remediation, but in recognition of the student’s unique situation, bringing unacceptable behavior to his/her attention, and support toward resolution. The non-specificity of behaviors that bring a student to attention might also explain why the offending actions observed during the dominantly classroom-based Years 1 and 2 did not differ from the behaviors observed during the dominantly clinical Years 3 and 4.

It is important to note that while students with responses in classes 1 and 2 progressed to subsequent ECNs at a lower rate than students in classes 3 and 4, the progression rate was not zero. This suggests that refraining from unprofessional behavior requires more than just recognition, good intentions, and insight. It is possible that some students in classes 1 and 2 are well intentioned but nevertheless struggle with issues of impulsivity, immaturity, ongoing personal challenges, or other contributions to subsequent lapses. More work is needed to explore such factors.

Ideally, students would receive direct feedback on their behavior (and other skills) from those with whom they work. The value of early-warning-systems such as ECNs and similar programs at other institutions derives in part from two phenomena [23]. First, there are limits to relying solely on direct feedback to students from those with whom they interact. Faculty, whether in classroom or clinical settings, often have limited scope of direct observation or may be hesitant to report isolated events either from insecurity, desire to avoid labeling a student, discomfort with confrontation, or fear of damaging the teacher–learner relationship [24,25]. Those who work transiently with learners may also hesitate to report isolated events but would feel more comfortable in doing so if they knew such behavior represented a pattern. Second, sharing performance information across courses is not routine, and if ‘isolated events’ go unreported, no pattern is ever detected [26]. Strengths of a graded-response ECN process include the ability to maintain a supportive and counseling-dominant environment until a pattern is recognized that justifies stronger action [27]. In this respect, our ECN program reflects the tiered approach of Hickson et al. with licensed physicians that emphasizes progression from a ‘cup of coffee’ conversation to more formal, structured intervention when warranted [28].

Threats to reliability of any classification process can exist if students respond with what they think is the right answer during the meeting. Such a phenomenon would result in misclassification from a higher class (3 or 4) to a lower one. This may have contributed to the finding that some students in the low-risk classifications (1 and 2) progressed to subsequent ECNs.

Our work supports the premise that ECNs can be a practical, objective and easy-to-implement reporting, and classification strategy. It additionally demonstrates the importance of student responses to an ECN. A process that identifies students at increased risk of repetitive problematic behaviors at the time of their first ECN creates an opportunity to intervene or interrupt patterns of behavior [27,29]. Several students in our study group were dismissed in part or in whole because of unprofessional behavior, so the ramifications of allowing problematic behaviors to go unrecognized or uncorrected can be substantial. Since this population may also be at higher risk for problems after graduation, interventions tailored to a student’s individual needs, including more intense observation, feedback, counseling, or leaves of absence, appear justified [30,31].

Most students in this study appeared to have had no conscious intent to be unprofessional, a gratifying result. It is possible that their low rate of progression may reflect personal growth, professional identity development, or simply the impact of students seeing that their behaviors were actually noticed, rather than the counseling provided through this program [23]. But since students in class 2 were clearly surprised by the professionalism norm they violated, the ECN can serve an educational function as well.

Any ECN system has limitations. Like all screening instruments, its sensitivity exceeds its specificity, and adjunctive information is needed to more clearly isolate the subset of students identified in the screening process most likely to benefit from intervention. Incorporating student reactions to ECN reports may provide that information and improve specificity. It is also reasonable to speculate that some of the reactions displayed by students in class 3 might actually be normal adaptive behavior. For example, when faced with a required task in life one deems unpleasant or unnecessary (such as navigating a poorly designed electronic medical record system), compliance ‘just to avoid sanctions’ (preferably with feedback to the designers) may be a learned functional or productively adaptive response. Yet, the rate of progression to subsequent ECNs in this group (36%) suggests this represents a high-risk group.

It is not clear why students with three or more ECNs were also more likely to experience adverse academic events. It is possible that they had additional underlying skill deficiencies that impacted their broader performance or that the same behavioral issues resulting in ECNs were deleterious to their academic preparation, such as rule-following or attention to clinical responsibilities. A small number of students acknowledged facing substantial personal challenges (life stressors, substance abuse, and/or mental health problems) with impacts ranging from mild distraction to substantial impairment, but they were not otherwise distinguishable by the ECN process. However, it seems clear that academic failures alone cannot be used as a reliable early warning system.

Cautions to the generalizability of these findings are warranted. At our institution, the ECN system does not operate in isolation. It is part of a broader initiative addressing professionalism which includes a Professionalism Charter for all faculty, students, and employees, frequent workshops on the subject, a biennial professionalism symposium, and support from leadership. A range of reporting opportunities for undesired behaviors is provided, including both formal and informal (and anonymous) options. This environment may mean our students and faculty are more sensitized or receptive to the program. Additionally, results are from a single school and student response classifications were judgments of a single author, a practice chosen to minimize exposure of confidential information and to maximize consistency of interpretation of student responses. However, classifications were purposefully designed to limited subjectivity; for example, class 1 was used only when the student’s initial response unambiguously acknowledged the inappropriateness of the behavior and acceptance of responsibility without qualification. Class 2 was used only when such acknowledgements were forthcoming by the end of the meeting. While re-review of a subset of blinded classifications by independent raters was included to strengthen the reliability of such judgments, misclassification may have occurred. Work to examine generalizability of this approach across raters and institutions is needed. A small number of classifications were based on a telephone or videoconference interview of the student rather than an in-person interview because of geographic separation between the parties. Absence of in-person interaction may have influenced the judgments being made. Finally, the follow-up period in the study did not extend through the entire medical school career of all students, so the number of subsequent ECNs or adverse academic events may be incomplete.

This analysis did not examine impact on future practice. While such results are powerful reflections of the impact of professional behavior on patient care and a physician’s career, medical schools also need surrogate measures which allow them to identify as early as possible, among all students who misstep, that critical subpopulation at highest risk of additional problems while enrolled [17,23,32]. Using professionalism reports like ECNs as a screening method in the way described offers schools another tool to help identify those students most in need of assistance. If the value of this approach to the use of ECNs is verified, they could also (with appropriate safeguards) become part of a ‘feed-forward’ system designed to alert future courses that additional attention to these skills in such students is appropriate. Examples of safeguards used at our institution include requiring student permission for such a process and providing information only to course directors who could select assignments tailored to the student’s needs—not to faculty who directly grade the student.

## Data Availability

None.

## Previous presentations

Abstracts of earlier versions of this work were presented at the

- 124th Annual Meeting of the Association of American Medical Colleges. Philadelphia, PA. November 2013.
- Association of Medical Education in Europe annual meeting. Glasgow, Scotland. August 2015.
- Learn, Serve, Lead meeting of the Association of American Medical Colleges. Boston, MA. November 2017.
